# Hydrological seasonality drives a predictable reversal in anthropogenic pollution dominance in mountain watersheds

**DOI:** 10.1016/j.isci.2026.116366

**Published:** 2026-06-15

**Authors:** Hui Yang, Yuhan Zhao, Xianglong Hou, Zhiying Li, Jiansheng Cao

**Affiliations:** 1 Key Laboratory of Agricultural Water Resources, Hebei Key Laboratory of Agricultural Water-Saving, Center for Agricultural Resources Research, Institute of Genetics and Developmental Biology, Chinese Academy of Sciences, Shijiazhuang, China; 2 School of Advanced Agricultural Sciences, University of Chinese Academy of Sciences, Beijing, China; 3 Institute of Geographical Sciences, Hebei Academy of Sciences, Shijiazhuang, China; 4 Hebei Technology Innovation Center for Geographic Information Application, Shijiazhuang, China; 5 O’Neill School of Public and Environmental Affairs, Indiana University Bloomington, Bloomington, IN, USA

**Keywords:** environmental science, pollution, hydrology

## Abstract

Seasonal hydrology can fundamentally reorganize the drivers of ecosystem function. Here, we uncover a seasonal reversal of pollution dominance (SRPD) mechanism in six monsoonal watersheds of China’s Taihang Mountains. By coupling the water quality index (WQI) with principal-component and redundancy analyses, we develop a diagnostic framework that decodes this dynamic. Human-intensive basins show significantly greater seasonal parameter volatility than forested ones. Cropland explains 90.9% of constrained variance in dry seasons, whereas impervious surfaces emerge as the key driver in wet seasons (75.1%). The derived SRPD axis explains 54.5%–91% of pollution variance. Spatial hotspots of seasonal degradation, identified via a ΔWQI (difference between wet-season and dry-season WQI) model, highlight the ZH and HTH watersheds as most vulnerable. Our framework transforms seasonal monitoring data into a quantifiable signal of shifting anthropogenic forcing, offering a tool for dynamic ecosystem assessment and seasonally adaptive management in monsoon-affected regions globally.

## Highlights

• Seasonal hydrology drives a reversal in pollution dominance
• Cropland dominates in dry season; impervious surfaces in wet season
• SRPD axis explains 54.5%–91% of pollution variance
• ΔWQI identifies ZH and HTH as the most vulnerable hotspots

## Graphical abstract

**Figure:**
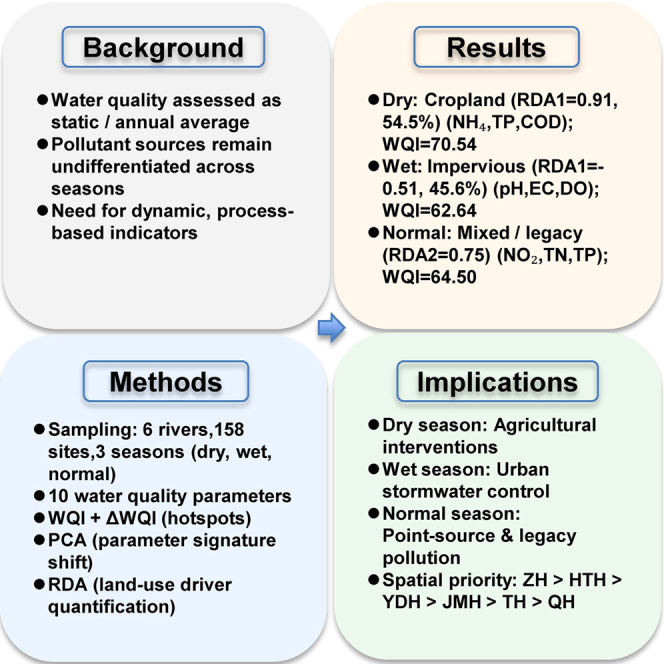

## Introduction

The dynamics of riverine ecosystems are fundamentally pulsed, driven by seasonal hydrology, which acts as a master variable reorganizing biogeochemical processes and pollutant transport.^1^ River water quality, a key integrator of watershed function, is, therefore, acutely sensitive to such seasonal forcing, ultimately determining its capacity to sustain aquatic ecosystems, agriculture, industry, and public health.^2^^,^^3^^,^^4^ For instance, the characteristics of dissolved organic matter and nutrient dynamics directly influence aquatic biodiversity and drinking water safety.^5^^,^^6^ However, pervasive anthropogenic pressures—including urbanization, agricultural expansion, and industrialization—have precipitated a widespread decline in water quality,^7^ a trend particularly acute in developing nations such as China.^8^^,^^9^ In China, over half of the population resides in rural areas where domestic wastewater is often released untreated,^10^ and expanding livestock farming frequently lacks sufficient waste treatment, exacerbating the problem.^11^ Compromised water quality, notably from chemical oxygen demand (COD) and ammonia nitrogen pollution,^12^ severely constrains agricultural efficiency, necessitating dilution water volumes equivalent to triple that of major national water transfer projects.^13^

To assess such impacts, the water quality index (WQI) has become an extensively utilized tool globally, synthesizing multiple parameters into a standardized score.^14^^,^^15^^,^^16^^,^^17^ Recent advancements even integrate machine learning to enhance WQI’s predictive accuracy.^18^ Nevertheless, a critical limitation persists—traditional applications often treat water quality as a static or annually averaged state,^19^ failing to capture the profound seasonal dynamics imposed by hydrological cycles. This obscures time-specific pollution sources and hinders the design of effective interventions.

This gap is especially critical in mountainous watersheds, which provide 40%–80% of freshwater resources for downstream settlements^20^ and are highly sensitive to land-use changes due to steep slopes accelerating pollutant transport.^21^ Seasonal rainfall patterns, with prolonged dry periods, exacerbate water scarcity and concentrate pollutants during low flow.^22^ The Taihang Mountain region, a vital ecological barrier and water source for the North China Plain,^23^ epitomizes these challenges. Over 60% of the plain’s rivers originate here, supplying 3.6 billion m^3^ annually.^24^ While significant ecological restoration has occurred since the 1980s, its impact on water quality remains uncertain,^25^^,^^26^ compounded by a lack of long-term monitoring data needed to understand pollutant migration and non-point source mechanisms.^27^ Current research in the region predominantly focuses on hydrology and vegetation,^28^^,^^29^ with systematic assessments of river water quality under combined natural anthropogenic drivers remaining scarce.^23^^,^^30^^,^^31^^,^^32^

A pivotal but unexplored aspect is the potential for a systematic seasonal reversal in the dominance of anthropogenic pollution sources (e.g., agricultural vs. urban) across hydrological phases. Quantifying this seasonal reversal of pollution dominance (SRPD) and decoding its underlying hydro-ecological mechanisms is essential to move beyond static snapshots toward process-informed, dynamic indicators. Therefore, this study aims to (1) mechanistically decode the SRPD phenomenon and its drivers across six typical mountain watersheds, and (2) develop an integrated diagnostic framework capable of capturing this dynamic to inform seasonally adaptive management strategies for sustainable water quality governance.

## Results

**Figure 1:**
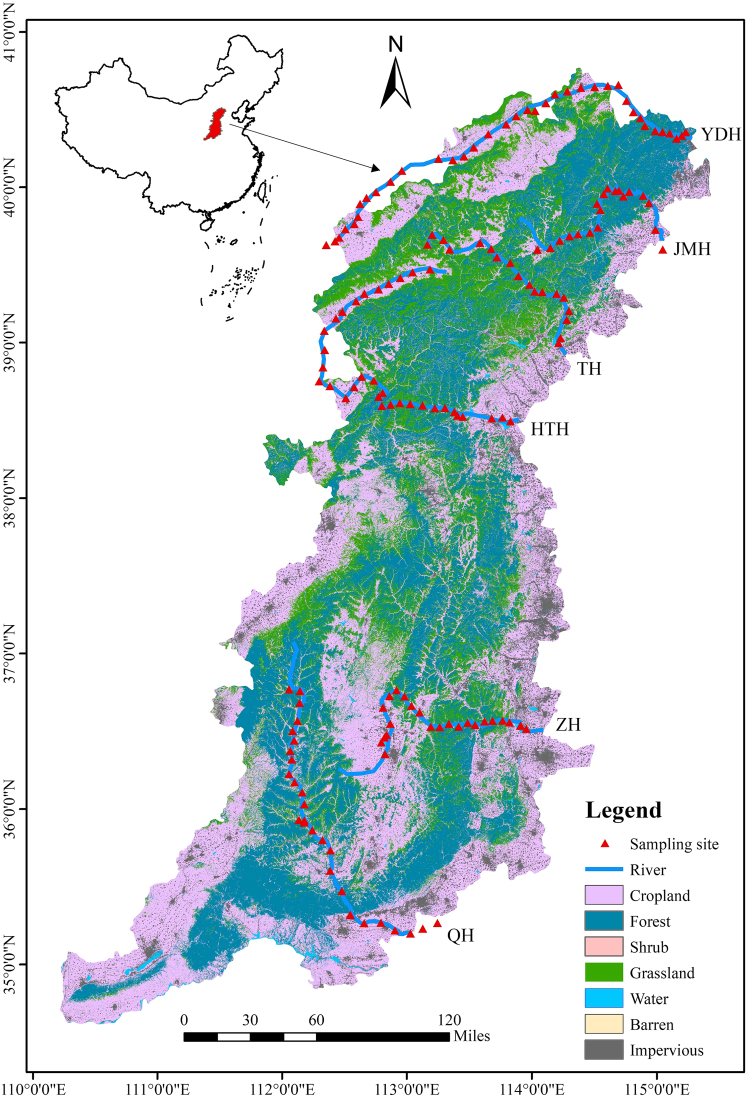
Location of the study area and sampling sites

The study area spans a 131,785 km^2^ and encompasses six major rivers—the Yongdinghe (YDH), Jumahe (JMH), Tanghe (TH), Hutuohe (HTH), Zhanghe (ZH), and Qinhe (QH)—flowing from west to east across the Taihang Mountain into the North China Plain (Figure 1). A total of 158 sampling sites were established along the mainstems.

### Spatiotemporal dynamics of WQI and parameters

**Figure 2:**
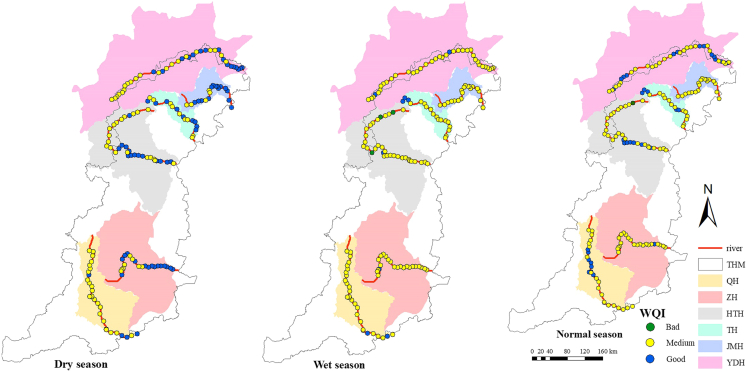
Spatiotemporal distribution of WQI values across six rivers in three sampling periods

The WQI showed a consistent “dry-season superiority” pattern across the six watersheds in our sampling year (Figure 2), serving as a baseline seasonal dynamic for this study. The mean WQI was highest in the dry season (70.54 ± 2.75), followed by the normal (64.50 ± 2.49) and wet seasons (62.64 ± 1.86), indicating a systematic decline in integrated water quality during high-flow periods.

As shown in Figure 2, in the dry season, WQI values across river sections ranged from medium to good. The JMH and ZH rivers exhibited predominantly good WQI values, with 73.7% and 65.2% of their sections, respectively, classified as good. The QH river showed mostly medium WQI values, with only a few sections rated as good. For the YDH, TH, and HTH rivers, good and medium WQI sections were evenly distributed, with good values concentrated in the middle and lower reaches.

During the wet and normal seasons, the number of sections with good WQI values decreased significantly, and some sections showed poor ratings. In the wet season, medium WQI values dominated, accounting for at least 80% of sections across all rivers. The TH river’s headwaters retained good WQI values, while sections of the HTH river were rated poor. In the normal season, the proportion of good WQI sections increased for the YDH river from 5.4% to 35.1%, that for the HTH river from 3.0% to 21.25%, and that for the QH river from 7.1% to 28.6%, while poor WQI sections in the HTH river decreased from 9.1% to 3.0%. Notably, all sections of the JMH river remained medium in both wet and normal seasons, and the ZH river showed no seasonal variation between the wet and normal seasons. Overall, WQI evaluations indicated that water quality in the Taihang Mountain rivers is best in the dry season, followed by the normal season, and poorest in the wet season.

### Spatiotemporal dynamics of water quality parameters

**Figure 3:**
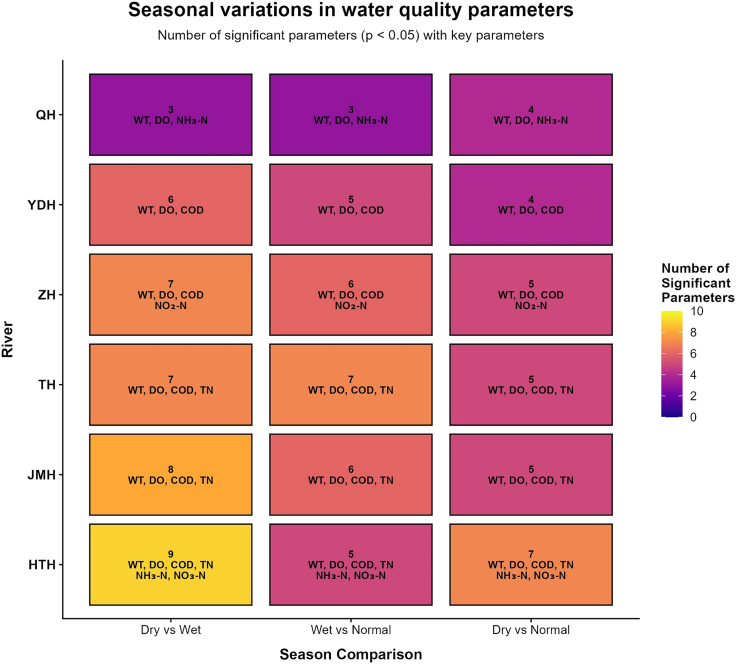
Summary of significant seasonal variations in key water quality parameters across the six rivers The number indicates the count of parameters (out of 10 measured) that showed statistically significant differences (*p* < 0.05) in each seasonal comparison.

Hydrological seasonality was the dominant force governing water quality dynamics across the six watersheds. Independent-samples *t* test analysis revealed that hydrological seasonality was the dominant force governing water quality dynamics, with the most pronounced shifts occurring between the dry and wet seasons. The sensitivity of each river to these seasonal changes, quantified by the number of parameters that varied significantly (*p* < 0.05), was closely linked to its anthropogenic exposure (Figure 3). The HTH river, draining a basin with intensive human activity, exhibited the highest volatility, with up to 9 parameters fluctuating significantly between dry and wet periods. In stark contrast, the QH river demonstrated the greatest stability, a likely consequence of its more forested headwaters and lower overall anthropogenic pressure.

While physical parameters like water temperature (WT) and dissolved oxygen (DO) varied consistently across all rivers, following predictable seasonal thermodynamics, the most telling changes were observed in chemical indicators of human activity. Parameters such as COD, total nitrogen (TN), and nitrate nitrogen (NO_3_-N) showed the strongest and most frequent significant variations, particularly between dry and wet seasons. This pattern is consistent with a seasonally dependent pollutant flushing mechanism, wherein monsoonal rainfall may mobilize accumulated contaminants. We interpret this as supportive evidence for the pollution dominance reversal explored in subsequent sections, while acknowledging that direct hydrological measurements would strengthen causal attribution.

**Figure 4:**
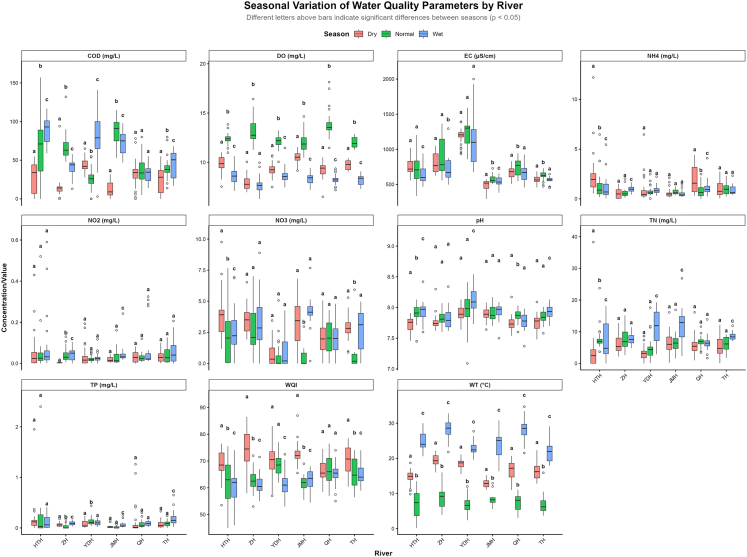
Seasonal distribution patterns of key water quality parameters across the six watersheds

Based on the overarching seasonal trends, a persistent spatial stratification was observed among the six rivers, which modulated their specific expression of the SRPD phenomenon. Boxplot visualization clearly revealed distinct seasonal patterns for the various parameters (Figure 4). Physical parameters such as WT and DO followed pronounced and consistent seasonal dynamics. Statistical testing confirmed that the WT showed extremely significant differences (*p* < 0.001) in all seasonal comparisons (dry vs. normal, dry vs. wet, and normal vs. wet) across every river (YDH, JMH, TH, HTH, ZH, and QH). Similarly, DO exhibited highly significant differences (*p* < 0.001) in nearly all seasonal contrasts for each river, with the only non-significant result being for ZH river between dry and wet seasons (*p* = 0.316).

Key anthropogenic indicators displayed decisive and often river-specific seasonal patterns. For instance, in the HTH river, TN was significantly higher during the dry season than in both normal (*p* = 0.005) and wet (*p* = 0.022) seasons. Conversely, COD showed a different dynamic, with significant seasonal differences observed in all three comparisons for rivers like HTH, JMH, YDH, and ZH (*p* < 0.001 for most), indicating a strong, yet varied, hydrological driver. These contrasting intra-river seasonal trends provide statistical evidence for the presence of distinct pollution sources (e.g., agricultural nitrogen versus organic/municipal COD) activated or mobilized by different hydrological conditions within the watersheds.

Spatial differentiation among the rivers remained evident when comparing their respective seasonal patterns. For electrical conductivity (EC), significant seasonal differences within rivers were less common but present, such as in TH river between dry and normal seasons (*p* = 0.04). Regarding pH, northern rivers like YDH and HTH showed highly significant decreases from dry to wet seasons (*p* < 0.001), while southern rivers like ZH and QH exhibited no significant seasonal pH shifts, underscoring a north-south gradient in the buffering capacity or influence.

Inter-river differences in seasonal behavior highlighted the spatial dimension of anthropogenic pressure. The HTH river demonstrated significant seasonal differences for nutrients like NO_3_-N (dry vs. normal: *p* < 0.001; dry vs. wet: *p* = 0.007), aligning with its agricultural character. Notably, COD seasonal variability was particularly pronounced in several rivers during the wet season, with JMH showing significant differences between dry and wet seasons (*p* < 0.001) and normal and wet seasons (*p* = 0.01), suggesting substantial wet-season flushing of organic pollutants even in forested basins.

The WQI effectively integrated these parameter shifts, revealing that most rivers (e.g., YDH, JMH, HTH, and ZH) experienced highly significant changes in the WQI between seasons, particularly between dry and wet periods (*p* < 0.001). This “spreading” of WQI values during the dry season underscores how low-flow conditions amplify the expression of underlying watershed characteristics, from stable, good quality in forested headwaters to more impacted conditions in agricultural and urban-influenced valleys.

### Seasonal restructuring of water quality parameter correlations

**Figure 5:**
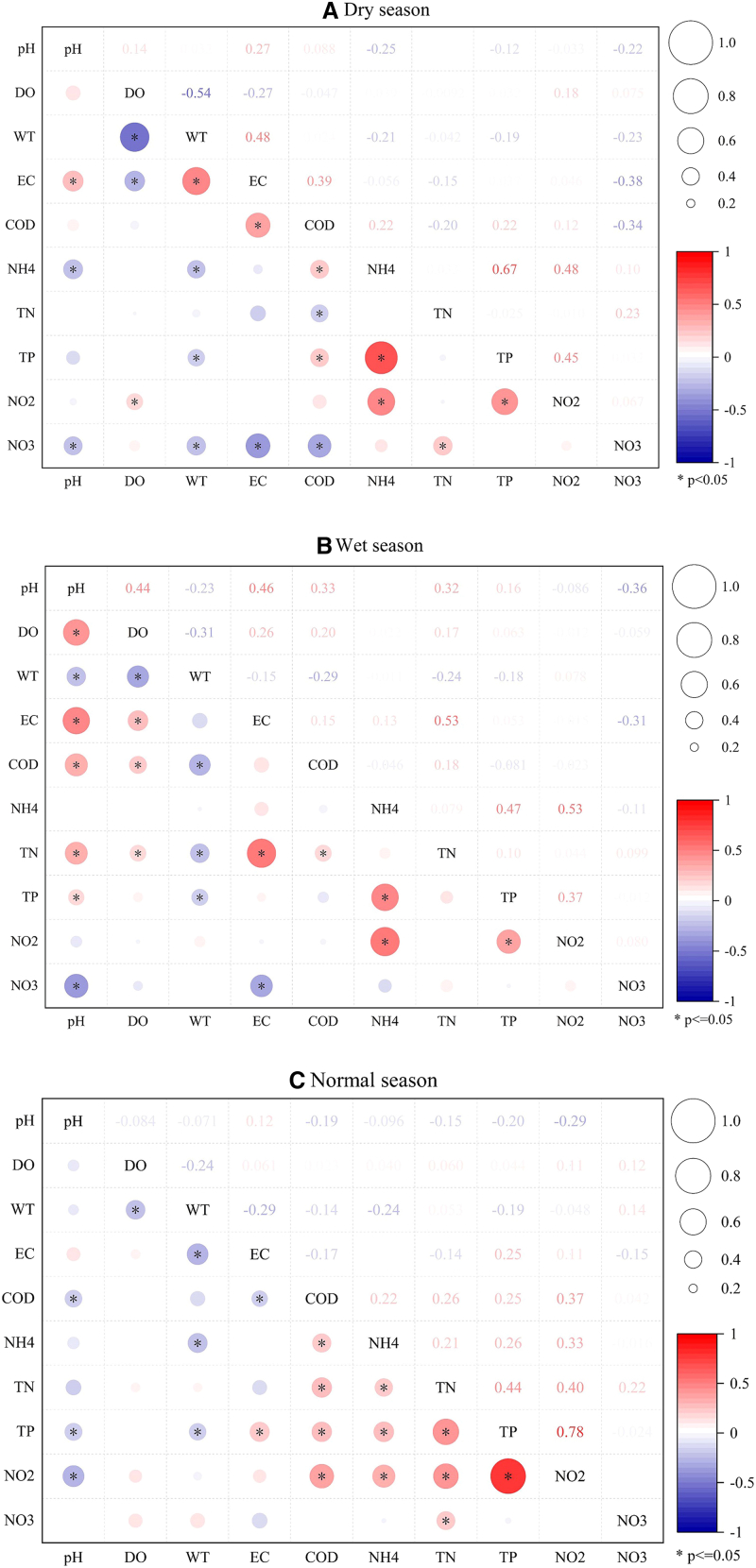
Seasonal correlation matrices of water quality parameters (A) Dry season. (B) Wet season. (C) Normal season.

Correlation analysis revealed distinct seasonal shifts in the interrelationships among water quality parameters, providing statistical evidence for the SRPD mechanism (Figure 5). All reported correlations were statistically significant at *p* < 0.05.

The dry season exhibited strong agricultural pollution signatures, with NH_4_-N forming a contamination cluster through significant positive correlations with TP (r = 0.67) and NO_2_-N (r = 0.48). The agricultural nutrient network extended through the correlation between NO_2_-N and TP (r = 0.45), indicating coupled nitrogen and phosphorus pollution from agricultural sources during low-flow conditions. Thermal and ionic influences were evident through the correlation between EC and WT (r = 0.48), while the strong negative correlation between WT and DO (r = −0.54) reflected thermal effects on oxygen solubility. Negative correlations between EC and NO_3_-N (r = −0.38) and between COD and NO_3_-N (r = −0.34) suggested complex nitrogen transformation pathways.

The wet season demonstrated a complex interplay of urban-agricultural mixed influences, with pronounced thermal regulation. The agricultural signature persisted through correlations between NH_4_-N and NO_2_-N (r = 0.53) and between NH_4_-N and TP (r = 0.47). Urban runoff characteristics were strongly evident in the correlation between EC and TN (r = 0.53) and between EC and pH (r = 0.46). The oxygen-alkalinity relationship emerged through the DO-pH correlation (r = 0.44), while nitrate exhibited negative correlations with pH (r = −0.36) and EC (r = −0.31), indicating acidification effects from nitrate sources. Thermal influences were widespread, with the WT showing negative correlations with DO (r = −0.31), COD (r = −0.29), TN (r = −0.24), and pH (r = −0.23), reflecting temperature-mediated regulation.

The normal season revealed a complex transitional pattern dominated by nitrogen-phosphorus coupling and thermal influences. The exceptionally strong positive correlation between NO_2_-N and TP (r = 0.78) represented the strongest relationship observed across all seasons, indicating tightly coupled nitrite and phosphorus dynamics during transitional flow conditions. Additional nitrogen-phosphorus linkages appeared through the correlation between TP and TN (r = 0.44) and between NO_2_-N and TN (r = 0.40), forming an integrated nutrient network. Thermal regulation was evident through multiple negative correlations—NO_2_-N with pH (r = −0.29), WT with EC (r = −0.29), WT with NH_4_-N (r = −0.24), and WT with DO (r = −0.24)—reflecting temperature-mediated effects on multiple water quality parameters.

Across all seasons, the correlation structures transitioned from tightly coupled agricultural pollution networks in dry season (maximum r = 0.67) to complex urban-agricultural-thermal mixed patterns in wet season (maximum r = 0.53), and finally to strongly coupled nitrogen-phosphorus relationships with thermal mediation in normal season (maximum r = 0.78). This progression quantitatively supports the SRPD framework, demonstrating that dry season water quality is governed by agricultural nutrient couplings, wet season conditions feature intricate interactions among agricultural sources, urban runoff, and thermal regulation, while normal season represents a phase of intense nitrogen-phosphorus interactions moderated by thermal regimes.

### Seasonal reversal of dominant water quality parameters

**Figure 6:**
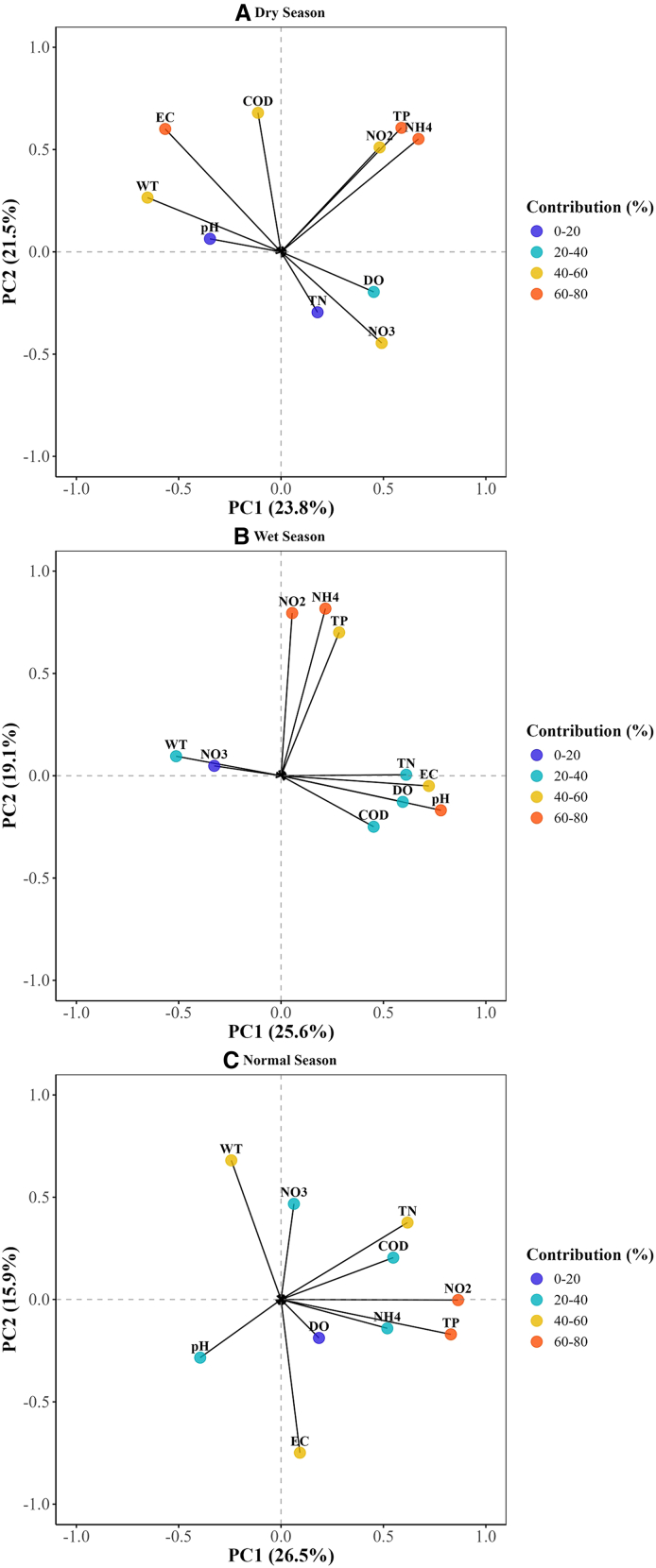
Principal-component analysis biplots of water quality parameters across three hydrological seasons (A) Dry season. (B) Wet season. (C) Normal season.

Principal-component analysis (PCA) revealed a fundamental restructuring of the dominant water quality parameters across the three hydrological seasons, quantitatively capturing the shifting pollution signatures (Figure 6). The cumulative variance explained by the first four principal components (PCs) (eigen values >1) was 69.89% for the dry season, 67.54% for the wet season, and 64.40% for the normal season, indicating that PCA could effectively capture the major patterns of water quality variation in each period.

Dry season patterns were characterized by a strong agricultural nutrient signature, with the first two PCs explaining 45.37% of the total variance (Figure 6A). PC1 (23.84%) was dominated by agricultural pollutants, showing high positive loadings for NH_4_-N (0.67), TP (0.59), and NO_2_-N (0.48). PC2 (21.53%) reflected a mix of ionic load from EC (0.60) and organic pollution from COD (0.68), with additional contributions from NH_4_-N (0.55) and TP (0.61). This clear separation highlights the concurrent yet distinct influences of agricultural nutrients and urban/organic pollution under low-flow conditions.

Wet season patterns exhibited a complete parameter restructuring, where PC1 (25.61%) shifted to urban runoff indicators, with strong positive loadings for pH (0.78), EC (0.72), and DO (0.59) (Figure 6B). Conversely, PC2 (19.11%) was overwhelmingly dominated by agricultural flushing signals, demonstrated by very high loadings for NH_4_-N (0.82), NO_2_-N (0.80), and TP (0.70). This orthogonal separation between urban-influenced PC1 and agriculture-dominated PC2 encapsulates the SRPD mechanism.

Normal season parameters revealed transitional complexity (Figure 6C), with PC1 (26.48%) capturing combined nitrogen transformation processes and phosphorus pollution, evidenced by extremely high loadings for NO_2_-N (0.86) and TP (0.83), alongside TN (0.62) and COD (0.55). PC2 (15.92%) primarily reflected thermal regulation through WT (0.68), with additional influence from NO_3_-N (0.47). This pattern suggests a transition period where point sources, accumulated pollutants, and natural thermal dynamics collectively govern water quality.

### Seasonal reversal in pollution drivers

**Figure 7:**
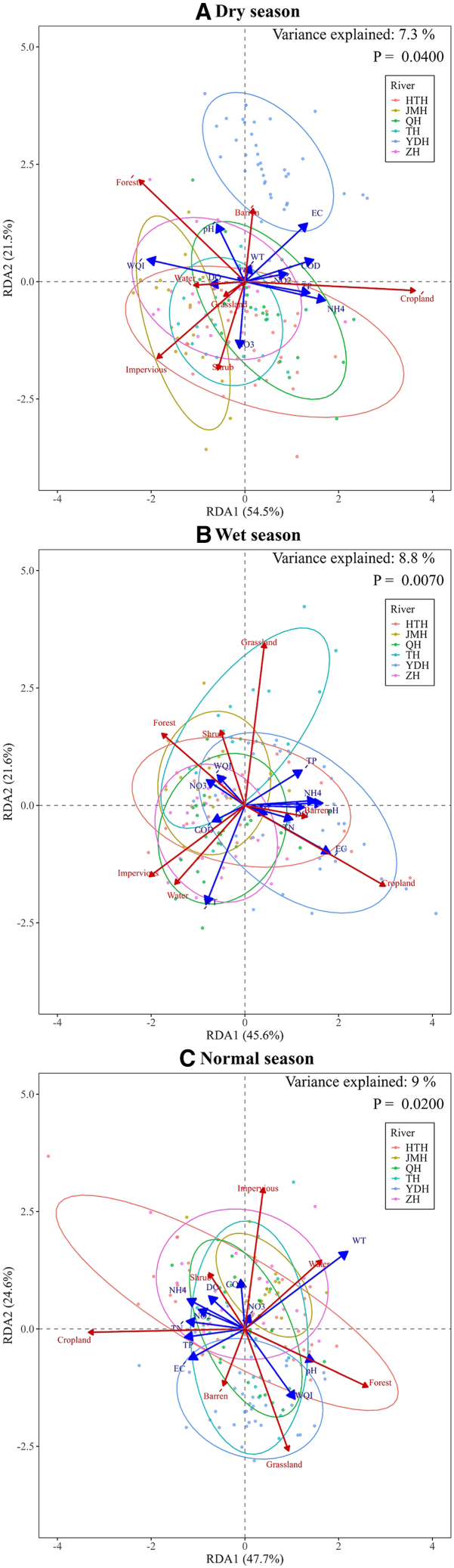
RDA ordination biplots showing land use-water quality interactions (A) Dry season. (B) Wet season. (C) Normal season.

Redundancy analysis (RDA) quantitatively revealed a distinct seasonal reversal in the primary land-use drivers of water quality (Figure 7). All seasonal models were statistically significant (*p* < 0.05), with land-use variables explaining 7.3%–9.0% of the total variance in water quality parameters.

During the dry season (Figure 7A), the model, though explaining a modest 7.3% of the total variance (*p* = 0.04), revealed an overwhelming dominance of cropland, which accounted for 54.5% of the constrained variance on the first axis (RDA1 loading = 0.91). The RDA1 axis served as a quantitative indicator of dominant land-use pressure, pointing to cropland as the key driver. This agricultural signature was manifested through strong positive associations with key nutrient parameters (NH_4_-N and TP) and organic pollution (COD), while the WQI exhibited a strong negative correlation with impervious surfaces.

A fundamental restructuring occurred in the wet season (Figure 7B), where the model’s explanatory power increased to 8.8% (*p* = 0.007). While cropland remained influential (RDA1 loading = 0.75), it now shared primary explanatory power on RDA1 (45.6% of constrained variance) with impervious surfaces (loading = −0.51), indicating a shift in the dominant pressure indicator toward a mixed agricultural-urban signal under high-flow conditions.

The normal season represented a transitional phase (Figure 7C), with the model explaining the highest proportion of variance (9.0%, *p* = 0.02). In this period, the influence of cropland on RDA1 (47.7% of constrained variance) reversed to a negative association (loading = −0.83), while impervious surfaces completed a stark seasonal reversal, emerging as a strong positive driver on the second axis (RDA2 loading = 0.75). This sequential shift from cropland to impervious surface dominance on the primary explanatory axis provides direct multivariate statistical evidence for the SRPD mechanism, capturing the hydrological phase-dependent nature of pollution drivers.

### Spatial hotspots of seasonal quality degradation

**Figure 8:**
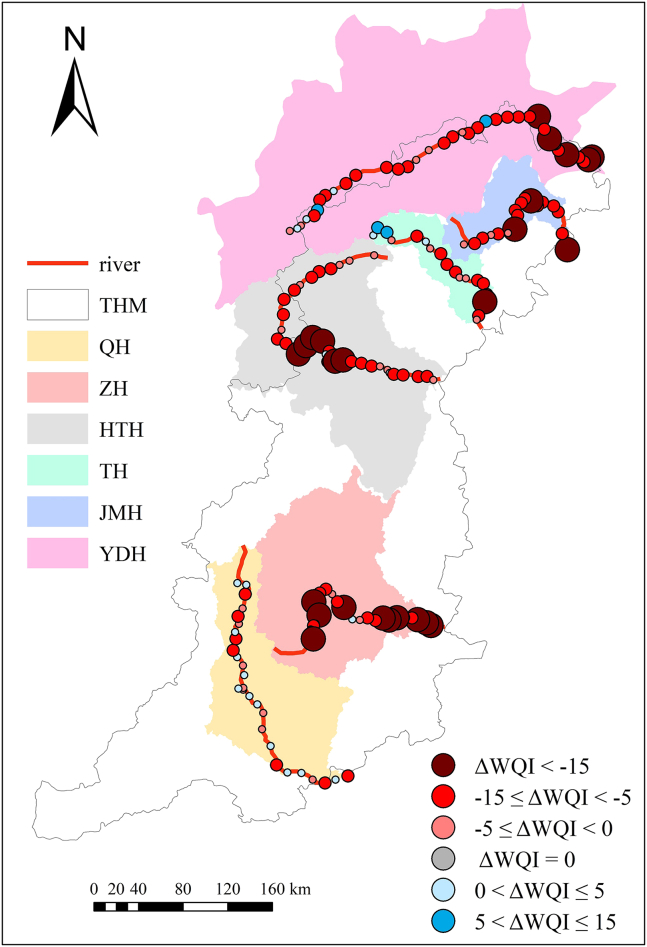
Spatial distribution of seasonal water quality degradation hotspots based on ΔWQI

The difference between wet-season and dry-season WQI (ΔWQI) was calculated to identify spatial hotspots of degradation (Figure 8). On the basis of ΔWQI classification, we identified 24 sampling sites (15.2% of total) experiencing severe degradation, designating them as highest-priority management areas.

The distribution of severe degradation hotspots revealed distinct watershed-specific vulnerability patterns, with the ZH watershed exhibiting the highest proportion of severely degraded sites (39.1%, 9 sampling sites), indicating extreme sensitivity to seasonal pollution pressures. The HTH watershed contained 6 severe degradation sampling sites (18.2% of its monitoring network), while the YDH and JMH watersheds showed 5 (13.5%) and 3 (15.8%) severely degraded sampling sites, respectively. The TH watershed demonstrated relative resilience, with only one severely degraded sampling site (5.6%), and the QH watershed showed complete resistance, with no sampling sites in the severe degradation category. Geospatially, these hotspots displayed distinct longitudinal patterns—YDH and ZH watersheds showed concentration in piedmont sections where rivers exit mountainous terrain, while HTH watershed exhibited predominant clustering in middle reaches, suggesting localized pollution accumulation zones. This piedmont distribution highlights the critical interface between mountain hydrology and plain-land anthropogenic pressures.

## Discussion

### The mechanism of SRPD

**Figure 9:**
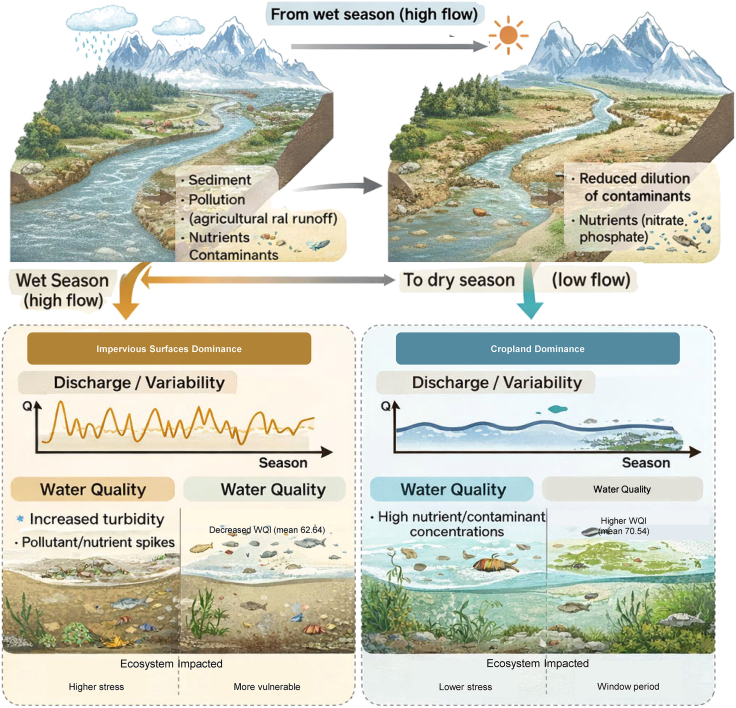
Conceptual diagram of the SRPD mechanism

The SRPD emerged as a pivotal mechanism governing water quality dynamics. This phenomenon emerges from the dynamic coupling of anthropogenic source availability and seasonally variable hydrological transport capacity.^33^ This SRPD mechanism (Figure 9) involves a reversal of dominant pollution sources between seasons.

Our results demonstrate that hydrological seasonality is not only a macro-scale driver of water quality variation but also modulates pollution patterns through differential basin sensitivity (Figure 3). Basins with intensive human activity (e.g., HTH) exhibited significant fluctuations in up to nine parameters between dry and wet seasons, reflecting high susceptibility to seasonal flushing and pollutant release. In contrast, basins with greater forest cover and lower anthropogenic pressure (e.g., QH) showed remarkable stability. This sensitivity gradient indicates that within the same climatic region, the inherent land-use composition and anthropogenic exposure of a watershed fundamentally shape the intensity and expression of the SRPD phenomenon. Therefore, deciphering the SRPD mechanism requires integrating seasonal hydrological forcing with basin-specific vulnerability, advancing from process identification to spatial prioritization.

The observed “dry-season superiority” in WQI (mean = 70.54) can be attributed to a combination of low-temperature suppression of microbial activity and reduced flow, which diminishes diffuse pollutant transport. Under these stable, low-flow conditions, our RDA results identify cropland as the dominant associated land-use factor (RDA1 loading = 0.91). This is consistent with the concentrated influence of irrigation return flows and the leaching of legacy nutrients (e.g., residual fertilizers) accumulated in soils, which become the principal determinants of water quality.^34^ The strong positive associations between cropland and nutrients (NH_4_-N and TP) and organic pollution (COD) in the dry season are direct manifestations of this agricultural signature.

The wet season triggers a mechanistic switch. Intense monsoon rainfall acts as a “pollution flush,” simultaneously activating two pathways. The first involves amplifying cropland non-point source pollution, leading to significant increases in nutrient concentrations, and the second is unlocking the potential of impervious surfaces (RDA1 loading = −0.51). Urban stormwater runoff efficiently scours and transports accumulated contaminants from streets and surfaces, leading to a surge of pollutants that can overwhelm a river’s assimilation capacity. The PCA results further corroborate this shift, showing a clear orthogonal separation between urban-influenced parameters (pH, EC, and DO) and agriculture-dominated signals (NH_4_-N, TP, and NO_2_-N) on different axes during the wet season (Figure 6B). This clear orthogonal separation between urban-influenced PC1 and agriculture-dominated PC2 visually encapsulates the SRPD mechanism, where different pollution sources are activated and transported via distinct pathways during high-flow conditions. This separation reflects the activation of distinct pollutant reservoirs under contrasting hydrological conditions.

The normal season represents a transitional phase where hydrological legacy effects prevail. The receding flow allows for the expression of point sources and the delayed flushing of accumulated urban pollutants, as indicated by the complete reversal of impervious surfaces to a strong positive driver on the second axis (RDA2 loading = 0.75). Concurrently, lower WTs can impair reaeration, potentially promoting anaerobic processes and internal nutrient loading, reflected in the PCA’s identification of nitrogen transformation processes (NO_2_-N and TN) as dominant factors.

The SRPD, elucidated by our RDA-PCA-WQI framework, emerges as a useful diagnostic indicator for monsoon-driven watersheds. It signals which anthropogenic source (agriculture vs. urban) may require prioritized management intervention in a given hydrological season.

### Land use interactions and spatial vulnerability

The manifestation of SRPD is influenced by land use interactions. Cropland exerted the most pervasive influence, yet its impact was spatially modulated by the configuration of other land types, particularly impervious surfaces. The antagonistic effect between cropland and impervious surfaces (r = −0.27), reflected in their negative joint contributions, highlights the spatial segregation of agricultural and urban landscapes in the Taihang Mountains, which fundamentally shapes how the SRPD manifests across different watersheds.^27^

**Figure 10:**
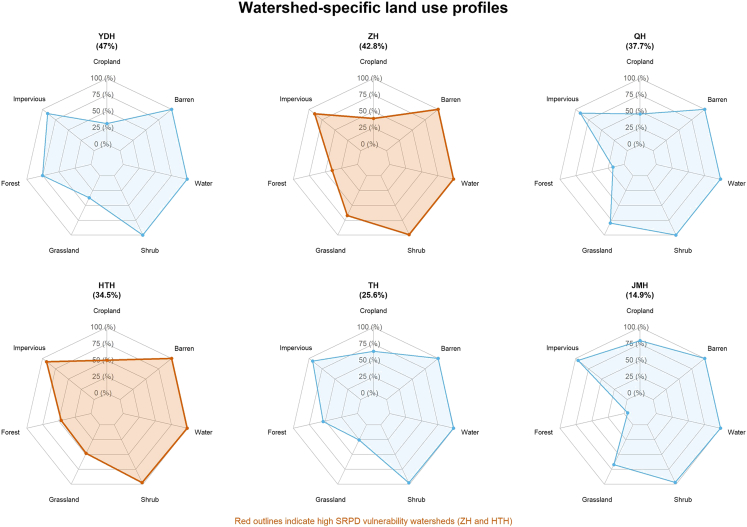
Proportional area distribution of different land-use types across six rivers

This spatial patterning is relevant for management. Our hotspot analysis (Figure 8) translates the mechanistic understanding of SRPD into actionable geography. The extreme vulnerability of the ZH watershed, followed by HTH, can be directly linked to their specific land-use compositions (Figure 10) and their location at the critical interface between mountain hydrology and piedmont anthropogenic pressures. The concentration of degradation hotspots in these piedmont zones underscores them as priority areas for intervention, where the “flushing” effect from upstream and the concentrated pollution from the plains converge. Thus, the interaction between cropland and impervious surfaces, quantified by variance partitioning and correlation, may provide a supplementary spatial indicator for anticipating watershed-specific vulnerability to the SRPD phenomenon.

### Broader transferability of the SRPD framework

The SRPD mechanism identified in this study is likely a generalizable phenomenon in regions subjected to strong seasonal precipitation pulses. While manifested as an agriculture-to-urban dominance shift in our monsoonal study area (Figure 9), the core principle—that different pollutant reservoirs (e.g., soil legacy nutrients and urban surface stockpiles) are activated and transported by distinct hydrological pathways—may translate to other climatic and hydrogeological settings. For instance, in snowmelt-dominated basins, a shift from dormant winter point sources (e.g., wastewater effluent under ice cover) to spring runoff-driven non-point sources (e.g., agricultural and urban runoff) might constitute a similar seasonal regime shift.^35^ In Mediterranean climates characterized by dry summers and wet winters, the contrast between dry season’s concentrated wastewater influence (due to low dilution capacity) and wet season’s agricultural runoff flushing could be a critical dynamic governing annual water quality patterns.^22^ Our diagnostic framework (WQI integrated with PCA and RDA) is specifically designed to detect such phase-dependent driver reversals from routine seasonal monitoring data. Applying this framework across diverse hydro-climatic settings could test the universality of SRPD-like dynamics, improve process-based models predicting how climate change might alter pollutant transport regimes,^7^ and ultimately foster a more general paradigm of seasonally adaptive environmental assessment that moves beyond static, annualized benchmarks.^19^ The transferability of the framework lies in its ability to transform multi-parameter, seasonal data into interpretable axes of variation (e.g., RDA1 as a season-specific composite pressure indicator), offering a reproducible approach for decoding hydrological phase-dependent ecosystem responses in human-impacted watersheds worldwide.

### Implications for seasonally adaptive watershed management

The demonstration of SRPD suggests a shift from static, uniform management toward dynamic and adaptive strategies. Our RDA-validated findings, which quantify the seasonal switch in dominant associated factors, alongside the spatial hotspot indicator (ΔWQI), suggest distinct priorities for each hydrological phase and specific watershed context.

For dry season, management could capitalize on the annual window of optimal WQI to implement targeted agricultural interventions. This includes optimizing fertilizer application timing to minimize legacy soil nutrients, constructing riparian wetlands to intercept and treat irrigation return flows, and maintaining vegetated buffers, with focused attention on high-vulnerability watersheds like ZH and HTH.

For wet season, strategies may need to pivot rapidly to address urban stormwater amplification. Key actions include implementing green infrastructure (e.g., permeable pavements and rain gardens) in identified ΔWQI piedmont hotspots of YDH and ZH to enhance infiltration and retention, alongside real-time control of combined sewer overflows to mitigate the contaminant “first flush.”

For normal season, a dual-target strategy may be required to address lingering legacy effects and point source pollution. Attention could turn to upgrading wastewater treatment plants while also managing the lingering effects of urban contaminant accumulation, with special attention to middle-reach sections in HTH that exhibit clustering of degradation hotspots.

Spatially, resources could be allocated proportionally to watershed vulnerability (ZH > HTH > YDH > JMH > TH > QH), a hierarchy directly suggested by our ΔWQI hotspot analysis. This may entail immediate integrated agricultural-urban runoff controls in ZH’s piedmont zones and middle-reach pollution interception in HTH, while the relative resilience of TH and QH watersheds may only require conservation-oriented management.

### Limitations of the study

Several limitations should be acknowledged. First, our analysis is based on one year of seasonal sampling (2023). Multi-year monitoring would be needed to assess interannual variability. Second, while the RDA models explained a modest proportion (7.3%–9.0%) of total water quality variance, other unmeasured factors (e.g., groundwater inputs and in-stream biogeochemical processes) also play important roles. Future studies should incorporate these additional variables to improve explanatory power. Third, while we infer hydrological drivers from seasonal contrasts, direct measurements of river discharge and rainfall intensity at each sampling site would strengthen causal attribution. Future research should integrate continuous hydrological monitoring to link SRPD dynamics explicitly to flow regimes. Fourth, while our WQI and ΔWQI calculations follow established protocols,^36^^,^^37^ we acknowledge that the hotspot classification may be sensitive to the chosen weighting scheme. Future studies should explore the robustness of ΔWQI-based hotspot identification by using alternative weighting approaches or unweighted aggregation methods. Fifth, sampling sites are located along river mainstems, and nearby sites may exhibit spatial autocorrelation due to hydrological connectivity. While our analysis treated the sites as independent observations, we acknowledge that this assumption may not fully hold. Future studies should explicitly test for spatial autocorrelation by using methods such as Moran’s I or incorporate spatial regression approaches to account for connectivity effects.

## Resource availability

### Lead contact

Requests for further information and resources should be directed to and will be fulfilled by the lead contact, Hui Yang (yanghui@sjziam.ac.cn).

### Materials availability

This study did not generate new unique reagents.

### Data and code availability

The data used in this study are all available from public resources that have been appropriately cited within the manuscript. This study did not generate any custom code. Any additional information required to reanalyze the data is available from the lead contact upon request.

## Acknowledgments

This research was financially supported by the Project for Innovative Research Group of the 10.13039/501100003787Natural Science Foundation of Hebei Province, China (grant numbers D2025503014), the 10.13039/501100001809National Natural Science Foundation of China (grant number 42371048), the Science and Technology Fundamental Resources Investigation Program, China (grant number 2022FY100104), the Hebei Academy of Sciences Basic Scientific Research Funds Pilot Project, China (grant numbers 2026PF03), and Science and Technology Planning Project of Hebei Academy of Sciences, China (grant numbers 26A07).

## Author contributions

Data curation, X.H.; methodology, H.Y.; investigation, X.H. and Y.Z.; formal analysis, H.Y.; validation, Z.L.; software, Y.Z.; writing – original draft, H.Y.; writing – review & editing, Z.L. and J.C.; supervision, Z.L. and J.C.; resources, Z.L. and J.C.; project administration, J.C.

## Declaration of interests

The authors declare no competing interests.

## STAR★Methods

### Key resources table

**Table:** 

REAGENT or RESOURCE	SOURCE	IDENTIFIER
**Deposited data**		

DEM data	https://www.gscloud.cn/	N/A
Land use/land cover (LULC) data	http://www.ncdc.ac.cn	N/A
Water quality data	https://nesdc.org.cn	N/A

**Software and algorithms**		

Arc GIS 10.5	https://www.arcgis.com	N/A
IBM SPSS Statistics 20	https://www.ibm.com/products/spss-statistics	N/A
R software (4.3.0)	https://www.r-project.org/	N/A
Origin 2025b	https://www.originlab.com	N/A
Microsoft EXCEL 2016	https://www.microsoft.com	N/A

### Method details

#### Study area

The Taihang Mountain (THM) (34°30′0″*N*-41°0′0″N, 110°0′0″E−115°30′0″E) is situated to the west of the North China Plain,^21^ serving as an important geographical boundary between the second and third topographic steps of China, and separating the Loess Plateau from the North China Plain.^31^ The elevation across the Taihang Mountain area ranges from 800 to 2000 m.^30^ Characterized by a typical warm temperate semi-humid and semi-arid monsoon climate zone, the region experiences an average annual temperature of 10°C and receives an average annual precipitation of 570 mm.^29^ Precipitation is highly seasonal, with 60–70% concentrated between July and September, often as heavy rainfall that exacerbates soil erosion. The average annual land surface evaporation is 470 mm, while water surface evaporation reaches 1100 mm.^38^^,^^39^

Cropland is the dominant land use pattern, covering 37.8% of the total area, followed by forestland (32.9%), grassland (19.7%), and impervious surfaces (8.3%). The remaining land use categories—water bodies, barren land, and other impervious areas—collectively account for less than 2% of the region (Figure 1). Typical vegetation includes sparse shrubland, deciduous broadleaved forest, coniferous forest, sedges, and meadow plants. Major crops cultivated in the valleys comprise wheat, millet, corn, potato, legumes, and rape.

#### Water sampling and physicochemical analysis

Following the technical specifications for surface water environmental quality monitoring (HJ91.2–2022), a total of 158 sampling sites were established along the mainstems of the six rivers. According to the guidelines, for natural river sections without obvious pollution outfalls, a single monitoring section (background section) is established at each sampling site. All sites in this study met this condition. The number of vertical sampling lines and sampling points was determined based on river width and water depth. Given that most rivers in our mountainous study area have widths less than 50 m and depths less than 5 m, we established one vertical sampling line at the thalweg and collected one sample at each site. Based on the guidelines, which emphasize representative sampling away from dead-water zones and discharge outfalls, we selected a target interval of approximately 10 km to balance coverage with field operability. Due to accessibility constraints in mountainous terrain, the actual distances between some adjacent sites varied locally from the target interval. The apparent variation in sampling density in Figure 1 reflects both these practical deviations and the influence of river sinuosity on map projection. Sampling was conducted during three hydrological seasons in 2023: the dry season (April 17–May 12), the wet season (August 14–31), and the normal season (November 6–20). The watershed area and number of sampling sites for each river are summarized in below Table.

**Table:** Watershed area and number of water sampling sites for each river

River name	YDH	JMH	TH	HTH	ZH	QH
Watershed area/10^4^ km^2^	4.4	0.51	0.45	2.4	2.6	1.3
Number of water sampling sites	37	19	18	33	23	28

Water samples were collected at a depth of 0.5 m below the surface using 500 mL polyethylene bottles, stored in darkness at 4°C, and transported to the Laboratory of Agricultural Resource Center, Chinese Academy of Sciences for analysis. Ten water quality indicators were measured: water temperature (WT), pH, dissolved oxygen (DO), electrical conductivity (EC), chemical oxygen demand (COD_Cr_), ammonia nitrogen (NH_4_–N), total nitrogen (TN), nitrite nitrogen (NO_2_-N), nitrate nitrogen (NO_3_-N), and total phosphorus (TP). WT, pH, EC, and DO were determined on-site using an Aqua TROLL 400 multi-parameter water quality sonde (*In-Situ*, USA). All storage, preservation, and analytical procedures followed national standards for water and wastewater testing methods; detailed methodologies and references are provided in below table.

**Table:** Methods and references for determination of water quality indices

NO.	Variable	Abbreviation	Units	Method	Reference	Weight (*P*_*i*_)
1	Water temperature	WT	°C	Portable water quality analyzer	GB13195-91	1
2	pH value	pH	/		HJ1147-2020	1
3	Dissolved oxygen	DO	mg/L		HJ506-2009	4
4	Electrical conductivity	EC	μS/cm		⟪Methods of Analysis for Water and Wastewater Monitoring⟫(Fourth Edition)	1
5	Chemical oxygen demand	COD_cr_	mg/L	Spectrophotometry	HJ/T 399-2007	2
6	Ammonia nitrogen	NH_4_–N	mg/L	Flow analyzer	HJ 536-2009	3
7	Total nitrogen	TN	mg/L		HJ 636-2012	4
8	Nitrite nitrogen	NO_2_-N	mg/L	Spectrophotometry	GB 7493-87	3
9	Nitrate nitrogen	NO_3_-N	mg/L		HJ/T 346-2007	3
10	Total phosphorus	TP	mg/L	Fully automated chemical analyzer	HJ 671-2013	4

#### Water quality index (WQI) calculation

To synthesize multi-parameter data into a comparable metric for assessing spatial and temporal trends, the weighted arithmetic Water Quality Index (WQI) was calculated. Ten physicochemical parameters—WT, pH, DO, EC, COD_cr_, NH_4_-N, TN, NO_2_-N, NO_3_-N and TP—were selected to construct the WQI. The formula is as follows^40^:

$$WQI=\frac{\sum_{i}C_{i}P_{i}}{\sum_{i}P_{i}}$$

where *i* ∈ [1, 10] corresponds to the ten parameters, *C*_i_ represents the normalized value, and *P*_i_ denotes the assigned weigh.^41^ Weights (*P*_i_) range from 1 to 4. Higher values indicate greater ecological significance.

The final WQI scores, ranging from 0 to 100, classify water quality into five grades: 0–25 (very bad), 26–50 (bad), 51–70 (medium), 71–90 (good), and 91–100 (excellent).^36^ The weights assigned to each parameter reflect their relative ecological significance based on established protocols.^36^^,^^37^ Specifically, parameters with higher weights (e.g., DO, TN, TP) are considered more critical indicators of water quality impairment. The normalization factors and weights for each parameter are detailed in below Table.

**Table:** Normalization scores and relative weights of water quality parameters

Variable	Weight (*P*_i_)	Normalization factor (*C*_i_)										
		100	90	80	70	60	50	40	30	20	10	0
WT^a^	1	21/16	22/15	24/14	26/12	28/10	30/5	32/0	36/-2	40/-4	45/-6	>45/<-6
pH^a^	1	7	7–8	7–8.5	7–9	6.5–7	6–9.5	5–10	4–11	3–12	2–13	1–14
DO^a^	4	≥7.5	>7	>6.5	>6	>5	>4	>3.5	>3	>2	≥1	<1
EC^a^	1	<750	<1000	<1250	<1500	<2000	<2500	<3000	<5000	<8000	≤12000	>12000
COD_Cr_^a^	3	<5	<10	<20	<30	<40	<50	<60	<80	<100	≤150	>150
NH_4_–N^a^	3	<0.01	<0.05	<0.1	<0.2	<0.3	<0.4	<0.5	<0.75	<1	≤1.25	>1.25
TN^b^	2	<0.1	<0.2	<0.35	<0.5	<0.75	<1	<1.25	<1.5	<1.75	≤2	>2
NO_2_-N^a^	2	<0.005	<0.01	<0.03	<0.05	<0.1	<0.15	<0.2	<0.25	<0.5	≤1	>1
NO_3_-N^a^	2	<0.5	<2	<4	<6	<8	<10	<15	<20	<50	≤100	>100
TP^a^	1	<0.2	<1.6	<3.2	<6.4	<9.6	<16	<32	<64	<96	<160	>160

a Weights and normalization factors from Kannel et al.^36^

b Weights and normalization factors from Sang et al.^37^

To identify spatial hotspots of degradation, the difference between wet-season and dry-season WQI (ΔWQI) was calculated for each sampling site. Sites were classified into seven categories based on ΔWQI: severe degradation (ΔWQI < −15), moderate degradation (−15 ≤ ΔWQI < −5), light degradation (−5 ≤ ΔWQI <0), no change (ΔWQI = 0), slight improvement (0 < ΔWQI ≤5), moderate improvement (5 < ΔWQI ≤15), and significant improvement (ΔWQI >15).

#### Land use and spatial data

Digital Elevation Model (DEM) data with a spatial resolution of 30 m were acquired from the ASTER GDEM dataset via the Geospatial Data Cloud (https://www.gscloud.cn/). Watershed boundaries were delineated using hydrology tools in ArcGIS 10.5 software. Land use/land cover (LULC) data for the year 2022, also at 30-meter resolution, were obtained from the National Cryosphere Desert Data Center. (http://www.ncdc.ac.cn). Field surveys confirmed that land use patterns remained largely unchanged between the LULC data collection year (2022) and the water quality sampling period (2023). To characterize proximal anthropogenic influences, land use data within a 500-m riparian buffer zone around each sampling site were extracted with Arc GIS 10.5.^4^

### Quantification and statistical analysis

Prior to analysis, all water quality parameters and WQI values were assessed for normality using the Kolmogorov-Smirnov test.^42^ Non-normally distributed parameters were log-transformed or square-root transformed as appropriate to meet the assumptions of parametric tests. Homogeneity of variances was verified using Levene’s test. Seasonal variations were analyzed using independent samples t-tests,^43^^,^^44^ while differences among six rivers across three sampling periods were evaluated via LSD (Least Significant Difference) post-hoc tests following one-way ANOVA.^37^ To account for multiple comparisons, we applied a conservative significance threshold (*p* < 0.01) where appropriate, and all reported *p*-values are provided in the text.

Redundancy analysis (RDA), a constrained ordination method, was performed to decode and quantify the directional relationships between land use composition and water quality parameters (including WQI) across watersheds. This method allows for the extraction of key explanatory axes (e.g., RDA1), which themselves serve as composite indicators of the dominant anthropogenic pressures in each season. Arrows pointing in the same direction indicate a positive correlation; the smaller the angle, the stronger the correlation.^45^

Principal Component Analysis (PCA) was employed to identify the dominant combinations of water quality parameters that distinguish the three hydrological seasons, thereby revealing the underlying structure of pollution signatures. Seasonal correlation analyses among water quality parameters were performed using Pearson correlation analysis in Origin 2025b software, with all reported correlations significant at *p* < 0.05.

Together, the WQI, ΔWQI, PCA, and RDA constitute an integrated diagnostic framework designed to synthesize multi-parameter data, identify dominant parameter signatures, and quantitatively attribute water quality variation to land-use drivers across distinct hydrological seasons.

All statistical analyses were performed using IBM SPSS Statistics 20, R software (version 4.3.0), and Microsoft Excel 2016.
